# Dissection of the genetic architecture of three seed‐quality traits and consequences for breeding in *Brassica napus*

**DOI:** 10.1111/pbi.12873

**Published:** 2018-01-25

**Authors:** Bo Wang, Zhikun Wu, Zhaohong Li, Qinghua Zhang, Jianlin Hu, Yingjie Xiao, Dongfang Cai, Jiangsheng Wu, Graham J. King, Haitao Li, Kede Liu

**Affiliations:** ^1^ National Key Laboratory of Crop Genetic Improvement Huazhong Agricultural University Wuhan Hubei China; ^2^ Southern Cross Plant Science Southern Cross University Lismore NSW Australia

**Keywords:** rapeseed (*Brassica napus*), genomic variation, genome‐wide association study (GWAS), seed‐quality traits, quantitative trait loci (QTLs), candidate genes

## Abstract

Genome‐wide association studies (GWASs) combining high‐throughput genome resequencing and phenotyping can accelerate the dissection of genetic architecture and identification of genes for plant complex traits. In this study, we developed a rapeseed genomic variation map consisting of 4 542 011 SNPs and 628 666 INDELs. GWAS was performed for three seed‐quality traits, including erucic acid content (EAC), glucosinolate content (GSC) and seed oil content (SOC) using 3.82 million polymorphisms in an association panel. Six, 49 and 17 loci were detected to be associated with EAC, GSC and SOC in multiple environments, respectively. The mean total contribution of these loci in each environment was 94.1% for EAC and 87.9% for GSC, notably higher than that for SOC (40.1%). A high correlation was observed between phenotypic variance and number of favourable alleles for associated loci, which will contribute to breeding improvement by pyramiding these loci. Furthermore, candidate genes were detected underlying associated loci, based on functional polymorphisms in gene regions where sequence variation was found to correlate with phenotypic variation. Our approach was validated by detection of well‐characterized *FAE1* genes at each of two major loci for EAC on chromosomes A8 and C3, along with *MYB28* genes at each of three major loci for GSC on chromosomes A9, C2 and C9. Four novel candidate genes were detected by correlation between GSC and SOC and observed sequence variation, respectively. This study provides insights into the genetic architecture of three seed‐quality traits, which would be useful for genetic improvement of *B. napus*.

## Introduction

Rapeseed (*Brassica napus* L.) was formed ~7500 years ago by allopolyploidy between ancestors of *B. rapa* and *B. oleracea*, with cultivation recorded in Europe during the Middle Ages (Chalhoub *et al*., 2014). Rapeseed was originally the major source for lamp oil in Europe by the 16th century, although it was also used as edible oil by poor people (Gupta and Pratap, 2007; Snowdon *et al*., 2007). Earlier unimproved rapeseed cultivars usually contained about 50% erucic acid in oil and 60–100 μmol/g glucosinolate in meal (Snowdon *et al*., 2007; Wittkop *et al*., 2009). The erucic acid property was exploited, accompanied by large increases in cultivation in Northern Europe, during the second industrial revolution of the 19th Century, as a high temperature lubricant for steam engines (Daun, 2011). However, high erucic acid in oil was latterly thought to lead to cardiac damage and related health problems (Snowdon *et al*., 2007). High glucosinolate content in meal used for feed following oil extraction reduces its palatability and has been identified as a causal factor for diseases such as goitrogenic hypertrophy and liver/kidney problems in livestock (Schmidt and Bancroft, 2011). Reduction in seed erucic acid (EAC) and glucosinolate content (GSC) has therefore been an important goal in modern rapeseed breeding, leading to rapid development of ‘double‐low’ quality rapeseed cultivars (canola) during the 1970s, in which the seed quality was modified to achieve low EAC and low GSC (Gupta and Pratap, 2007). Notably, rapeseed (canola) has been the second largest oil crop after soya bean over the past 30 years and now provides 13.0%–16.0% of the world's vegetable oil production (https://www.ers.usda.gov/).

Oils stored in the embryos of seeds are important sources of fatty acids for human nutrition. Seed yield and seed oil content (SOC) have been identified as the most significant traits determining oil yield and thus driving crop improvement in rapeseed. It is estimated that a 1.0% increase in SOC is equivalent to an increase of 2.3%–2.5% in seed yield for oil production (Wang, 2004). Thus, increasing SOC is critical to meeting demand for edible oil and an important long‐term goal in rapeseed breeding. For example, the average oil content of cultivars developed in China between 2006 and 2010 was 43.25%, representing an increase of 5.33% compared with that in 2001–2005, with gains in the average SOC of rapeseed cultivar understood to have continued to increase in China by ongoing genetic improvement (Hu *et al*., 2017).

Due to their agricultural and economic importance, it is crucial to understand the genetic basis of these traits, and especially to identify more favourable alleles or target genes, which will enable plant breeders to deploy advanced cultivars having low EAC and GSC but high oil yield. Extensive studies by traditional quantitative trait locus (QTL) mapping, genome‐wide association studies (GWASs) and molecular techniques have already been carried out to dissect the genetic basis of these three seed‐quality traits. EAC has consistently been found to be controlled by two major QTLs on chromosomes A8 and C3 (Harper *et al*., 2012; Li *et al*., 2014; Qiu *et al*., 2006; Qu *et al*., 2017). In addition, EAC also appears to be affected by several other minor QTLs in *B. napus* (Qiu *et al*., 2006; Qu *et al*., 2017). Two *Brassica* genes, *BnaA08g11130D* and *BnaC03g65980D* in the *B. napus* reference genome that are homologous to *Arabidopsis fatty acid elongase 1* (*FAE1*) (Chalhoub *et al*., 2014), have been identified to be responsible for the two major QTLs controlling seed EAC, and causal mutations within these genes have been validated (Han *et al*., 2001; Wang *et al*., 2010b; Wu *et al*., 2008). *FAE1* encodes a 3‐ketoacyl‐CoA synthase that sequentially extends fatty acid chain lengths from C18 to C20 and C22 in the fatty acid biosynthetic pathway (Han *et al*., 2001).

Glucosinolate content has consistently been shown to be controlled by three major QTLs on chromosome A9, C2 and C9 (Harper *et al*., 2012; Howell *et al*., 2003; Li *et al*., 2014; Lu *et al*., 2014; Qu *et al*., 2015). Three orthologs of the *Arabidopsis MYB28* gene co‐localize with these QTLs and have been proposed as the candidate genes underlying GSC variation in rapeseed (Chalhoub *et al*., 2014; Harper *et al*., 2012; Lu *et al*., 2014). *MYB28* is a transcription factor that controls the biosynthesis of aliphatic glucosinolate (Gigolashvili *et al*., 2007). *MYB28* orthologs, *Bra035929* on *B. rapa* chromosome A9 and *Bo2g161590* on *B. oleracea* chromosome C2, had been identified in the two diploid ancestor of *B. napus*. However, these two *MYB28* orthologs are absent in the *B. napus* reference genome of ‘Darmor‐*bzh*’ (Chalhoub *et al*., 2014). Deletion of *MYB28* orthologs on A9 and C2 has provided confirmatory evidence of their causative role in reducing GSC in rapeseed (Harper *et al*., 2012). With the release of genome sequence for *Brassica* species, some additional candidate genes inferred to be involved in glucosinolate biosynthesis have also been identified (Lu *et al*., 2014). However, associations between GSC variation and sequence variation of most candidate genes have not been confirmed.

Rapeseed SOC is also affected by numerous QTLs (Delourme *et al*., 2006; Li *et al*., 2014; Liu *et al*., 2016; Sun *et al*., 2012; Zhao *et al*., 2012), with fourteen *Arabidopsis* orthologous lipid‐related genes been found at six QTLs (Zhao *et al*., 2012) and four rapeseed candidate genes at four QTLs differentially expressed between high‐ and low‐oil parents (Sun *et al*., 2012). The availability of the reference genome sequence now makes it possible to identify and resolve additional QTLs and candidate genes for SOC. GWAS based on ultra‐high‐density variant maps has been successful in identifying novel genes, networks and even functional variants in humans (Iotchkova *et al*., 2016), rice (Yano *et al*., 2016) and maize (Xiao *et al*., 2017). We therefore carried out a GWAS based on millions of polymorphisms that effectively saturate the *Brassica napus* genome.

In this study, 238 rapeseed cultivars representing a global distribution were re‐sequenced to identify comprehensive genomic variations within *B. napus*. GWAS was performed to identify loci associated with three seed‐quality traits including EAC, GSC and SOC, using a large set of high‐density SNPs/INDELs identified in an existing association panel of 189 diverse rapeseed inbred lines. Furthermore, we sought candidate genes underlying association loci and investigated functional variations in each candidate gene to identify those most likely to be responsible for each trait. This deep dissection will enhance our understanding of the genetic architecture of these major agronomic traits and provide informative clues for rapeseed breeding.

## Results

### Genomic variation for allotetraploid *B. napus*

**Figure 1:**
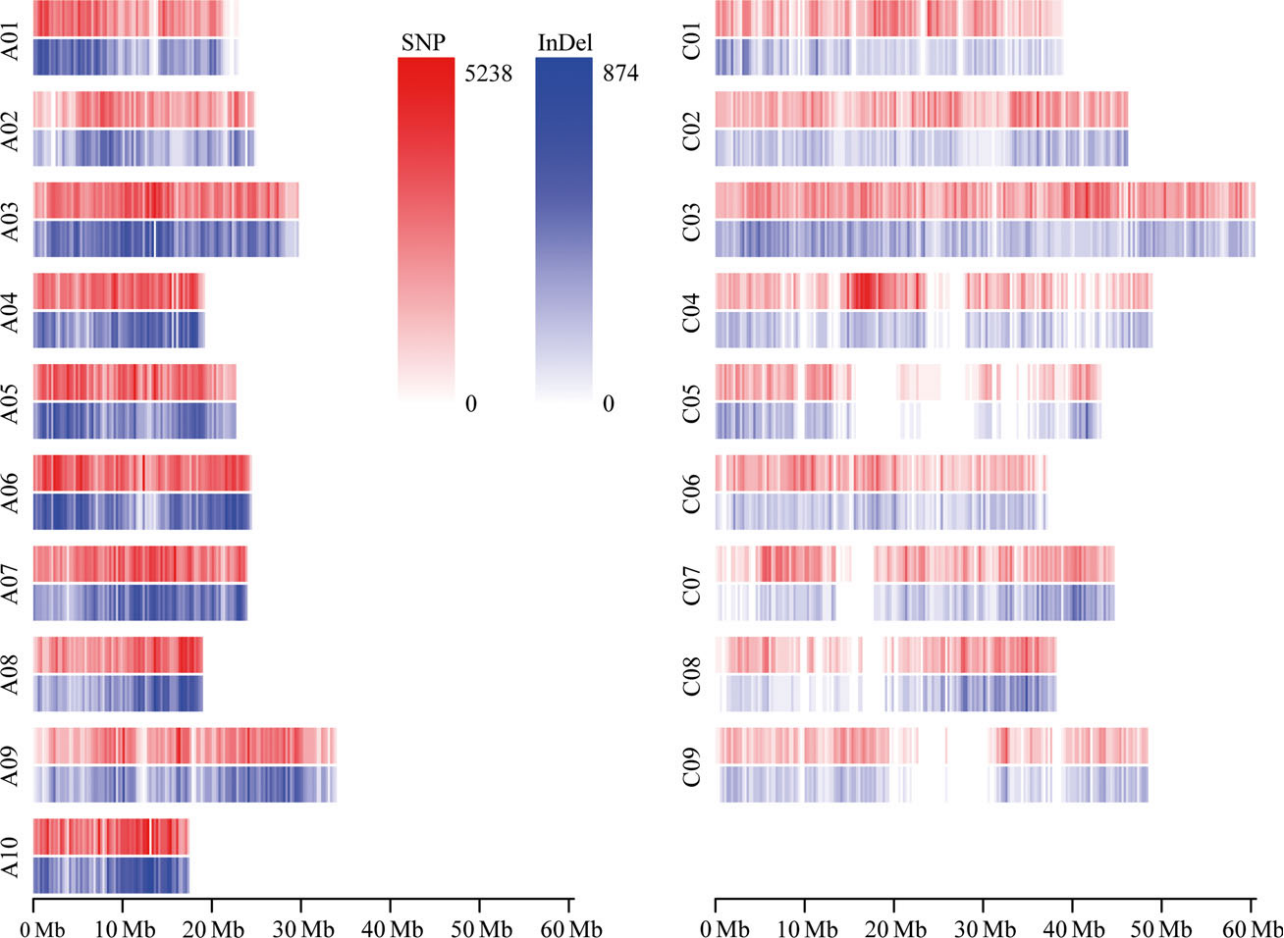
Heatmap for distribution of genomic variations in rapeseed. The number of SNPs and INDELs was calculated by 100‐kb sliding window across each chromosome.

To characterize genomic variation in the cultivated *B. napus* genepool, whole‐genome resequencing data were generated for 238 diverse rapeseed cultivars or inbred lines, which were collected from all the main rapeseed production regions around the world (China: 139, Europe: 56, Canada: 18, Australia: 13, Japan: 4 and unknown geographic origins: 8) (Table S1). A total of 1.98 Tb sequence data (9.9 billion paired‐end reads) was obtained, ranging from 3.95 to 35.21 Gb, with an average of 8.31 Gb for each line, equivalent to 7.35‐fold *B. napus* genome (~1.13 Gb, Chalhoub *et al*., 2014) (Table S1). The sequence reads for each line were aligned to the v4.1 draft reference genome of variety ‘Darmor‐*bzh*’. The mean genome coverage for each line was 78.1%, ranging from 60.3% to 89.4%. The coverage depth in each line ranged from 2.2‐ to 20.7‐fold, with a mean of 5.8‐fold (Table S1). A total of 10.7 million nonsingleton SNPs and 1.4 million nonsingleton INDELs were identified based on alignment to the reference genome. These SNPs and INDELs were comprised of interhomoeologue polymorphisms (IHPs), hemi‐SNPs/INDELs and simple SNPs/INDELs in the allotetraploid *B. napus*. After filtering SNPs and INDELs that had a high rate of heterozygosity and missing genotypes, a set of high‐quality simple variants was identified that included 5 696 903 SNPs and 768 336 INDELs. Of these, 4 542 011 SNPs and 628 666 INDELs were distributed on the 19 chromosomes of *B. napus* with a density of 7.65 and 1.09/kb, respectively (Figure 1; Table S2). The remaining variants were distributed on the nonanchored random scaffolds and were excluded from further analysis. The number and density of SNPs and INDELs varied dramatically between the A and C subgenomes (Figure 1; Table S2). The density of SNPs and INDELs in the A subgenome was 9.95 and 1.55/kb, about twice that found in the C subgenome (5.35 and 0.61/kb for SNPs and INDELs, respectively).

To gain insights into the potential effects of variants, all SNPs/INDELs detected were functionally annotated, based on the ‘Darmor‐*bzh*’ gene model v5.0 (Table S3). Most SNPs (65.01%) were located outside of genic regions. Of these, 44.28% were located in intergenic regions and 20.73% were located within the 1 kb upstream/downstream regions of genes. Of those in genic regions, 667 326 (14.69%) were located within introns, which is less than that in exons (17.15%). Moreover, 307 655 (6.77%) SNPs represented nonsynonymous substitutions, 2809 (0.06%) altered splicing junctions, 3757 (0.08%) induced gain of stop codon, and 918 (0.02%) induced loss of stop codon (Table S3). These SNPs may have significant effects on gene function. Most of the INDELs (65.69%) were also located outside of genic regions. Of these, 35.62% were located in intergenic regions, and 30.07% were located within 1 kb upstream/downstream regions of genes. Among the INDELs in genic regions, 150 990 (24.02%) were located within introns, which is significant higher than that in exons (3.62%). Moreover, 13 928 (2.22%) INDELs had a significant effect on amino acid sequences. Of these, 11 370 (1.81%) induced frameshift mutations, 2018 (0.32%) changed splicing junctions, 424 (0.07%) induced gain of stop codon, and 116 (0.02%) induced loss of stop codon (Table S3).

### Phenotypic variation

Extensive phenotypic variations for EAC, GSC and SOC were observed in the association panel of 189 inbred lines in different environments (Table 1; Figure S1). Based on the best linear unbiased prediction (BLUP) analyses, EAC had a wide range of variation with a 1370.5‐fold difference, whilst GSC varied 10.9‐fold between lines with lowest and highest values in the panel (Table 1; Figure S1). SOC had a relatively reduced range of phenotypic variation with a 1.4‐fold difference encompassing all lines (Table 1; Figure S1). These data are consistent with the known genetic basis and domesticated selection pressure for the different traits. ‘Double‐low’ rapeseed (canola) cultivars are defined as those produce less than 2% EAC and less than 30 μmol/g GSC (https://www.canolacouncil.org/). In the association panel, 121 inbred lines (64.0%) had EAC lower than <2% and 91 inbred lines (48.1%) had GSC lower than 30 μmol/g. Normality test also revealed that EAC and GSC had non‐normal distributions across all environments (Table 1). These results are consistent with EAC and GSC being subject to strong selection in modern rapeseed breeding. SOC showed a non‐normal distribution across all environments, with more lines skewed towards higher SOC (Table 1; Figure S1), indicating that SOC had also been under strong and more consistent selection in rapeseed breeding. The broad‐sense heritabilities (*H* ^2^) of EAC (99.88%), GSC (98.67%) and SOC (92.17%) were very high in each environment (Table 1). This suggests that all were stably inherited, although it appears that SOC is, to some extent, more sensitive to environmental factors.

**Table 1:** Phenotypic variation, variance components and broad‐sense heritability for all traits

Traits	Environments	Range (Min–Max)	Mean ± SD	‐Log_10_(*P*)^b^	ANOVA^c^				Heritability (*H* ^*2*^)^d^
					$\delta_{G}^{2}$	$\delta_{e}^{2}$	$\delta_{E}^{2}$	$\delta_{\mathrm{GE}}^{2}$	
EAC	2011	0.01–53.91	14.08 ± 20.04	18.06	401.37	0.49			99.96
	2012	0.01–55.95	14.6 ± 20.65	17.53	426.02	1.14			99.91
	All^a^	0.04–54.82	14.19 ± 20.15	18.07	406.71	0.8	0.02	0.72	99.88 (99.84–99.91)
GSC	2009	12.27–147.17	51.43 ± 36.22	12.41	1321.94	27.09			99.32
	2010	14.32–166.17	58.35 ± 42.08	12.98	1746.75	63.14			98.81
	2011	13.01–131.27	50.30 ± 33.28	12.45	1111.06	14.55			99.57
	All	13.78–150.19	53.5 ± 36.54	12.74	1354	35.44	15.26	42.9	98.67 (98.29–98.96)
SOC	2009	32.69–47.75	41.22 ± 2.60	1.78	6.1	1.49			92.47
	2010	29.5–46.25	40.12 ± 2.62	3.37	5.44	2.83			85.22
	2011	29.62–45.62	39.87 ± 2.88	4.57	7.51	2.78			89.02
	2012	28.34–44.32	38.18 ± 2.89	2.55	6.84	3.24			86.37
	All	31.65–44.99	39.76 ± 2.31	4.07	5.84	2.59	1.51	1.12	92.17 (90.41–93.53)

a The BLUP values of traits across all environments.

b *P* values of the Shapiro–Wilk test.

c All variances including δ_G_^2^, δ_E_^2^ and δ_GE_^2^ were significant at *p *<* *0.001.

d Broad‐sense heritability of each traits and the 95% confidence intervals for broad‐sense heritability across environments are indicated in parentheses.

### Associated loci and their genetic effects

**Figure 2:**
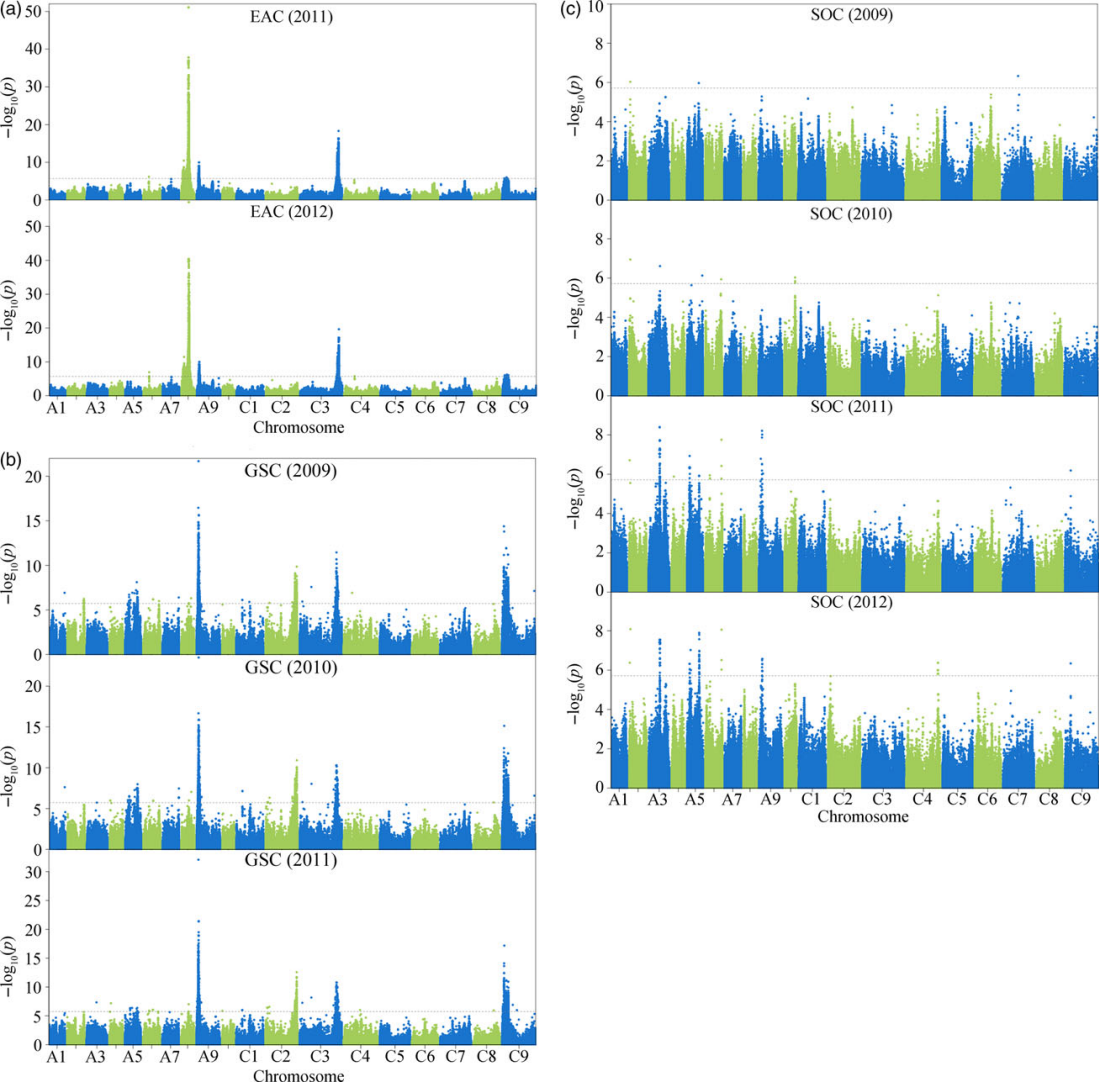
Genome‐wide association studies of three seed‐quality traits. Manhattan plots for EAC (a), GSC (b) and SOC (c) across multiple environments. ‐log_10_(*p*) values are plotted against position on each chromosome. Dashed grey lines indicate the genome‐wide significance threshold.

GWAS for all three traits was performed using the mixed linear model (MLM). A total of 72 loci were identified to be significantly associated with these traits (*P *<* *1.9 × 10^−6^) across all environments (Figures 2 and S2; Table S4). Six loci were associated with EAC, of which five located on chromosomes A6, A8, A9, C3 and were consistently detected in both environments (years 2011 and 2012), with the remaining locus on C4 only detected in a single environment (year 2012) (Figure 2a; Table S4). The genetic basis of GSC is clearly more complex than that of EAC, with 49 loci identified. Of these, more than half (31 loci, 63.27%), distributed on chromosome A1, A3, A5, A6, A8, A9, C1, C2, C3, C8 and C9, were consistently detected in multiple environments (Figure 2b; Table S4). Seventeen loci were associated with SOC, of which almost half (8 loci, 47.06%), distributed on A2, A3, A5, A6, A9 and C9, were consistently detected in multiple environments (Figure 2c; Table S4). The high proportion of loci that detected in multiple environments for all three traits reflects their high broad‐sense heritability.

**Figure 3:**
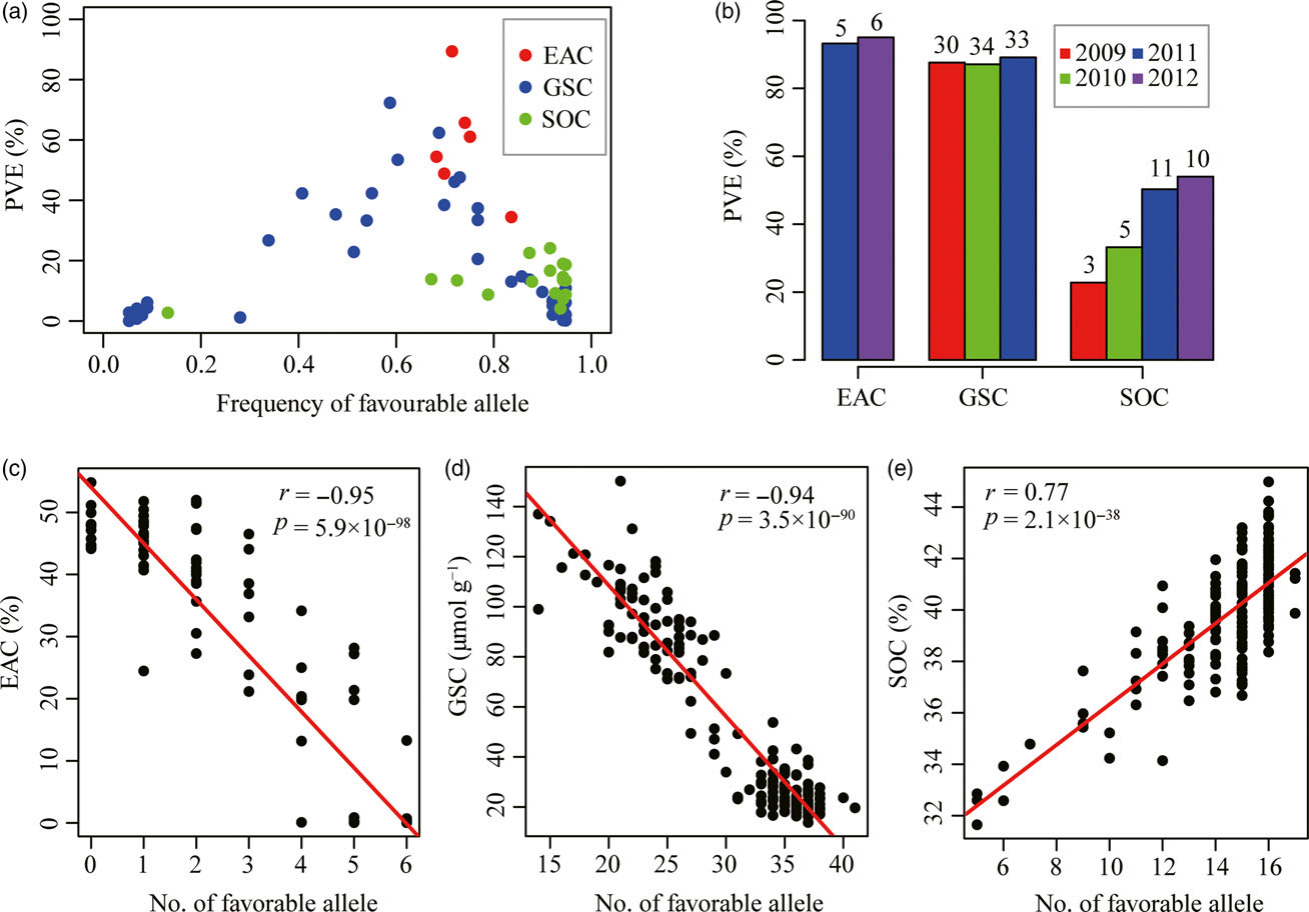
Characterization of PVE and favourable allele of associated loci for three traits. (a) The frequencies of favourable alleles and corresponding PVE. The favourable alleles of each loci for EAC and GSC were that reducing EAC and GSC in seeds, respectively. The favourable alleles of each loci for SOC were that increasing SOC. (b) Total PVE of all significant loci for three traits across each environment. Numbers on top of each bars indicated the number of loci detected in each year. (c‐e) Correlation between favourable allele number and EAC, GSC and SOC, respectively.

The genetic effects of all 72 associated loci were analysed further, with the peak SNP/INDEL selected to represent each associated locus. The phenotypic variation explained (PVE) by the associated loci ranged from 0.1% to 89.4% (Figure 3a). The six associated loci for EAC have PVEs ranging from 34.4% to 89.4% with a mean of 59.0%. The 49 loci for GSC have PVEs ranging from 0.1% to 72.3%, with a mean of 15.8%. The PVE of seventeen loci for SOC ranged from 2.7% to 24.1%, with a mean of 13.2%.

The combined contribution of all associated loci for each trait was further estimated in different environments (Figure 3b). The combined loci associated with EAC explained more than 90% of the phenotypic variation. For this trait, five loci were associated in 2011 (accounting for 93.2% of phenotypic variation) and six in 2012 (95.0%). For GSC, 30 loci were associated in 2009 (accounting for 87.6% of phenotypic variation), 34 in 2010 (87.1%) and 33 in 2011 (89.1%). For SOC, three loci were detected in 2009 (22.8%), five in 2010 (33.2%), 11 in 2011 (50.3%) and ten in 2012 (54.0%). These results demonstrate that the combined contribution for all three traits, especially for SOC, increased incrementally with the number of associated loci identified among different environments.

The frequencies of favourable alleles were also analysed for the three traits. At all six loci associated with EAC, the favourable alleles that reduce EAC had frequencies higher than 0.68, with an average of 0.74 in this panel (Figure 3a). For GSC, the average frequency of favourable alleles that reduce GSC at all associated loci was 0.62, of which 30 loci (61.3%) had frequencies higher than 0.6 (Figure 3a). For SOC, the frequencies of favourable alleles that increase SOC at all associated loci were higher than 0.67 (except for snp1448880 on A9), with an average of 0.85 (Figure 3a). This suggested that these favourable alleles had been under positive selection in the process of modern rapeseed breeding for improvement of seed quality, resulting in reduced seed erucic acid and glucosinolate content and increased oil content. To better understand the cumulative effect of associated loci, the relationship between number of favourable alleles and phenotypic variance was analysed for each trait. In the association panel, seed erucic acid and glucosinolate contents are negatively correlated with the number of favourable alleles (*r *=* *−0.95, *P *=* *5.9 × 10^−98^ for EAC and *r *=* *−0.94, *P *=* *3.5 × 10^−90^ for GSC; Figure 3c, d). In individual inbred lines, EAC and GSC reduce as the number of favourable alleles increases. In contrast, SOC is positively correlated to the number of favourable alleles (*r *=* *0.77, *P *=* *2.1 × 10^−38^; Figure 3e), suggesting that exploiting the additive effects of these loci by pyramiding favourable alleles is an efficient way to improve seed oil content in rapeseed.

### Candidate genes underlying associated loci

#### Erucic acid content (EAC)

**Figure 4:**
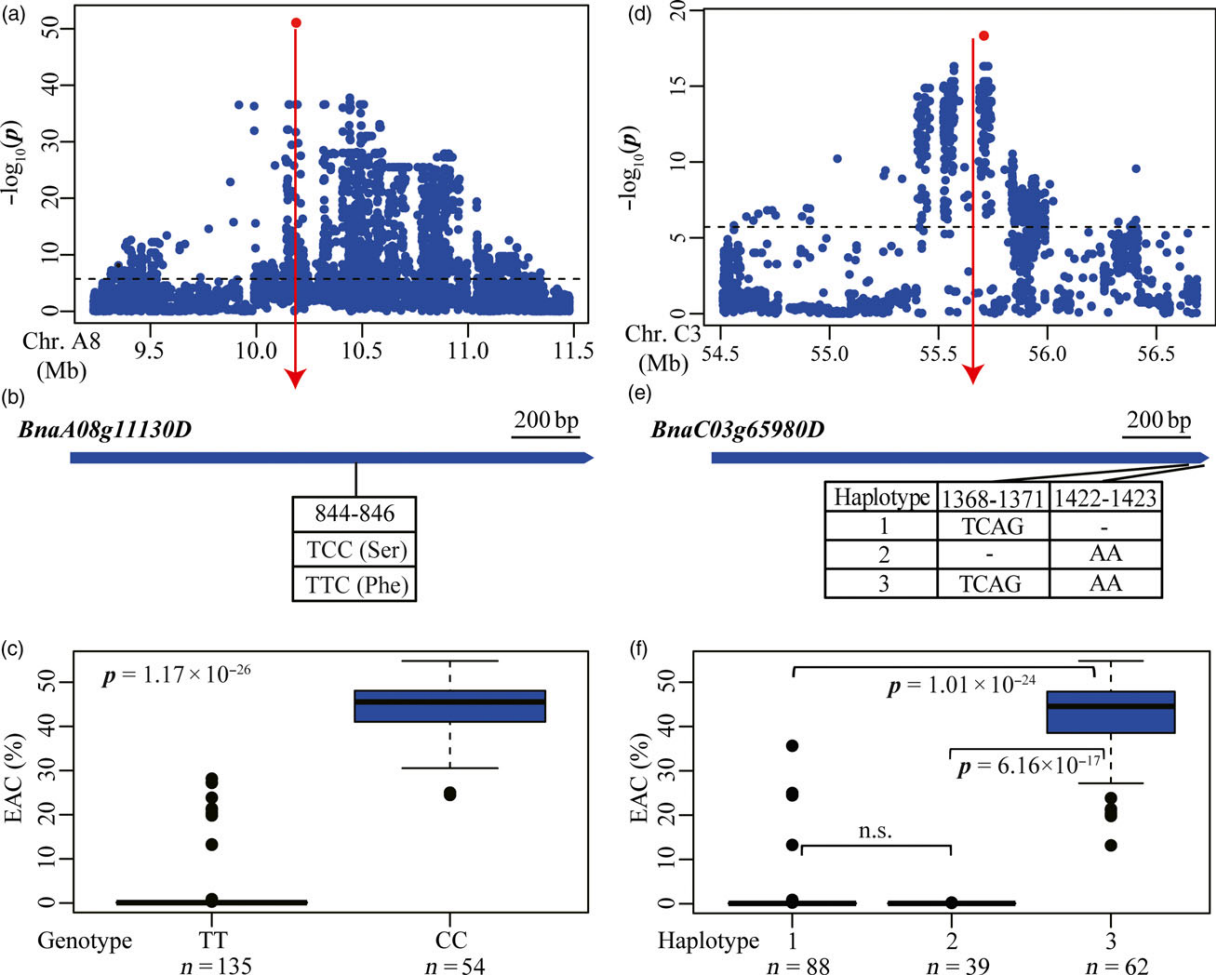
Associated loci and candidate genes for EAC on chromosome A8 and C3. (a, d) Regional Manhattan plot surrounding the peak signals on chromosome A8 (a) and C3 (d) in 2011. Red dot indicates the peak signals snp1348001 (a) and snp2429000 (d). Dashed line represents the significance threshold. (b, e) Exon structure and functional variations of *BnaA08g11130D* (b) and *BnaC03g65980D* (e). Numbers indicate the positions of open reading frame from translation start site. (c, f) Boxplots for EAC based on the genotypes of *BnaA08g11130D* (c) and haplotypes of *BnaC03g65980D* (f). Differences between the genotypes or haplotypes were analysed by Wilcoxon rank‐sum test. n.s. represents not significant.

To gain further insights into the genetic basis of EAC, candidate genes were sought in the genomic regions underlying the peak SNPs. Five candidates were found underlying the loci on A8, A9, C3, C4 and C9 (Table S5), with the peak signal snp1348001 on A8 significantly associated with EAC (Figue 2a). This SNP located in the coding region of *BnaA8.FAE1* (*BnaA08g11130D*) (Figure 4a; Table S5), which has previously been identified as the key gene in erucic acid synthesis in *Brassica* species (Han *et al*., 2001). Notably, the peak signal snp1348001 (C/T) was the exact causal SNP at the 845‐bp position of *BnaA8.FAE1* from the translation start site (Figure 4b), which results in the substitution of serine (Ser) with phenylalanine (Phe) at the 282th amino acid in low EAC cultivars, and prevents catalytically active conformation of FAE1 proteins in yeast cells (Han *et al*., 2001). The inbred lines with a TT genotype contain much lower erucic acid than those having a CC genotype in our panel (*P *=* *1.17 × 10^−26^) (Figure 4c). A peak SNP (snp2429000, *P *=* *4.68 × 10^−19^) was found 24 kb from another *fatty acid elongase* gene *BnaC3.FAE1* (*BnaC03g65980D*) on chromosome C3 (Figure 4d, Table S5). Within the coding region of *BnaC3.FAE1*, two deletions, a 4‐bp (TCAG) deletion at nucleotides 1368–1371 and a 2‐bp (AA) deletion at nucleotides 1422‐1423 from the translation start site, were identified in our panel (Figure 4e). These two deletions each resulted in a premature stop codon and thus a truncated protein, which can eliminate the enzymatic activity of *BnaC3.FAE1* and result in lack of production of erucic acid in yeast cells (Wu *et al*., 2008). Three haplotypes for *BnaC3.FAE1* were identified (Figure 4e). As expected, inbred lines carrying the functional haplotype 3 (TCAG/AA) showed significantly higher erucic acid content than lines carrying haplotype 1 (TCAG/‐, *P *=* *1.01 × 10^−24^) and haplotype 2 (‐/AA, *P *=* *6.16 × 10^−17^) (Figure 4f). Moreover, no difference in EAC was detected between inbred lines of haplotype 1 and haplotype 2 (Figure 4f). Remarkably, these results suggest that these two deletions in *BnaC3.FAE1* independently contributed to the reduction in EAC for *B. napus*. The identification of these two known genes *BnaA8.FAE1* and *BnaC3.FAE1* provides strong evidence that GWAS using high‐throughput markers in our panel has been efficient in helping us to identify candidate genes associated with EAC. In addition, three other candidates *BnaA09g07080D* (*BnaA9.ACX2*), *BnaC04g16670D* (*BnaC4.CYP94C1*) and *BnaC09g08890D* (*BnaC9.KCS9*) were found at the loci on A9, C4 and C9 (Table S5). These genes are orthologs of *Arabidopsis* genes encoding acyl‐CoA oxidase (ACX2), cytochrome P450 (*CYP94C1*) and 3‐ketoacyl‐CoA (KCS9), which are expected to be involved in long chain fatty acid biosynthesis (http://www.arabidopsis.org/).

#### Glucosinolate content (GSC)

**Figure 5:**
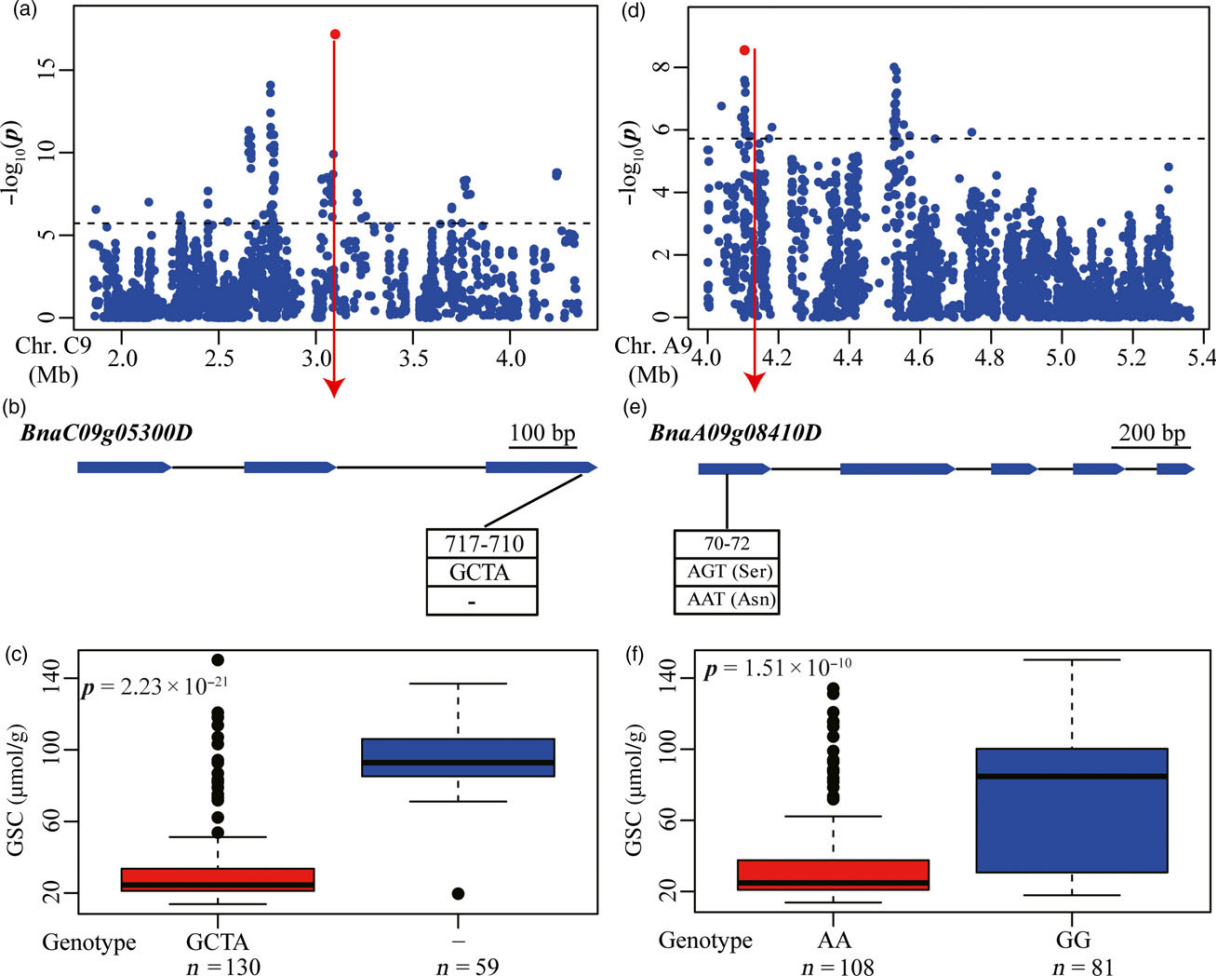
Associated loci and candidate genes for GSC on chromosome C9 and A9. (a, d) Regional Manhattan plot surrounding the peak signals on chromosome C9 (a) and A9 (d) in 2011. Red dot indicates the peak signal indel479620 (a) and indel240140 (d). Dashed line represents the significance threshold. (b, e) Exon–intron structure and functional variation of *BnaC09g05300D* (b) and *BnaA09g08410D* (e). Numbers indicate the positions of open reading frame from translation start site. (c, f) Boxplots for GSC based on the genotypes of *BnaC09g05300D* (c) and *BnaA09g08410D* (f). Differences between the genotypes were analysed by Wilcoxon rank‐sum test.

We also attempted to discover candidate genes in the genomic regions underlying all significant loci of GSC. This resulted in identification of 27 candidate genes involved in glucosinolate biosynthesis and breakdown at fifteen associated loci distributed on A2, A5, A6, A9, C1, C2 and C9 (Tables S5 and S6). The peak signal INDEL479620 (*R* ^2^ = 62.4%) was located within *BnaC9.MYB28* (*BnaC09g05300D*), an orthologous gene of *Arabidopsis MYB28*, on chromosome C9 (Figure 5a). The confirmed indel479620 (TAGC/‐, frameshift) was located in the third exon of *BnaC09g05300D* (*BnaC9.MYB28*) (Figures 5b and S3). The inbred lines containing TAGC had lower glucosinolate content than those with a TAGC deletion (*P *=* *2.23 × 10^−21^), further suggesting that variation in this gene was correlated with GSC (Figure 5c). The peak signal snp1438406 (*R* ^2^ = 72.3%) and indel338983 (*R* ^2^ = 53.4%) on A9 and C2, respectively, each was identified to be located within 100 kb of an orthologous gene of *Arabidopsis MYB28* (Figure S4). These three *MYB28* genes on A9, C2 and C9 co‐localized with the three major QTLs for GSC, and simultaneous deletions of the two *MYB28* genes on A9 and C2 were associated with reduced seed glucosinolate content in rapeseed (Harper *et al*., 2012). These results indicate the robustness of candidate identification for GSC in our study.

We also identified a number of candidate genes underlying the significant loci that had not been reported previously. The peak signal indel240140 on chromosome A9 was 27 kb apart from *BnaA09g08410D* (*BnaA9.APK1*) and 78 kb apart from *BnaA09g08470D* (*BnaA9.BGLU38*), which were considered as the most likely candidate genes (Figure 5d; Table S5). The homologs of these two genes in *Arabidopsis* (*APK1* and *BGL38*) are involved in co‐substrate pathways of glucosinolate biosynthesis and glucosinolate breakdown, respectively (Sønderby *et al*., 2010; Wittstock and Burow, 2010). No variations were identified in the gene *BnaA09g08470D*. However, a confirmed nonsynonymous SNP (G/A) was observed in the first exon of *BnaA09g08410D* (*BnaA9.APK1*) (Figures 5e and S3). The inbred lines carrying the AA genotype had lower GSC than lines carrying the GG genotype (*P *=* *1.51 × 10^−10^), suggesting that the variation in this gene was associated with GSC (Figure 5f). These observation demonstrated that *BnaA09g08410D* (*BnaA9.APK1*) is most likely to be the candidate gene for the locus represented by peak indel240140. All other 22 candidate genes were similarly analysed. This led to the identification of three additional candidate genes that affected GSC, including *BnaA05g23340D* (*BnaA5.NSP1*), *BnaA06g31890D* (*BnaA6.MYB118*) and *BnaA09g01260D* (*BnaA9.AOP3*) at the associated loci represented by snp846562, snp1064558 and snp1431371, respectively (Table S5; Figure S5). All observed variations in these gene regions were confirmed by sequencing of PCR product (Figure S3). Based on the functions of their orthologous genes in *Arabidopsis*,* BnaA9.AOP3*,* BnaA6.MYB118* and *BnaA5.NSP1* are likely to be involved in the pathways of aliphatic glucosinolate biosynthesis, benzenic glucosinolate biosynthesis and glucosinolate breakdown (Table S6). It appears likely that these genes are responsible for the peak signals detected and represent causal genes for variations of GSC in *B. napus*.

#### Seed oil content (SOC)

**Figure 6:**
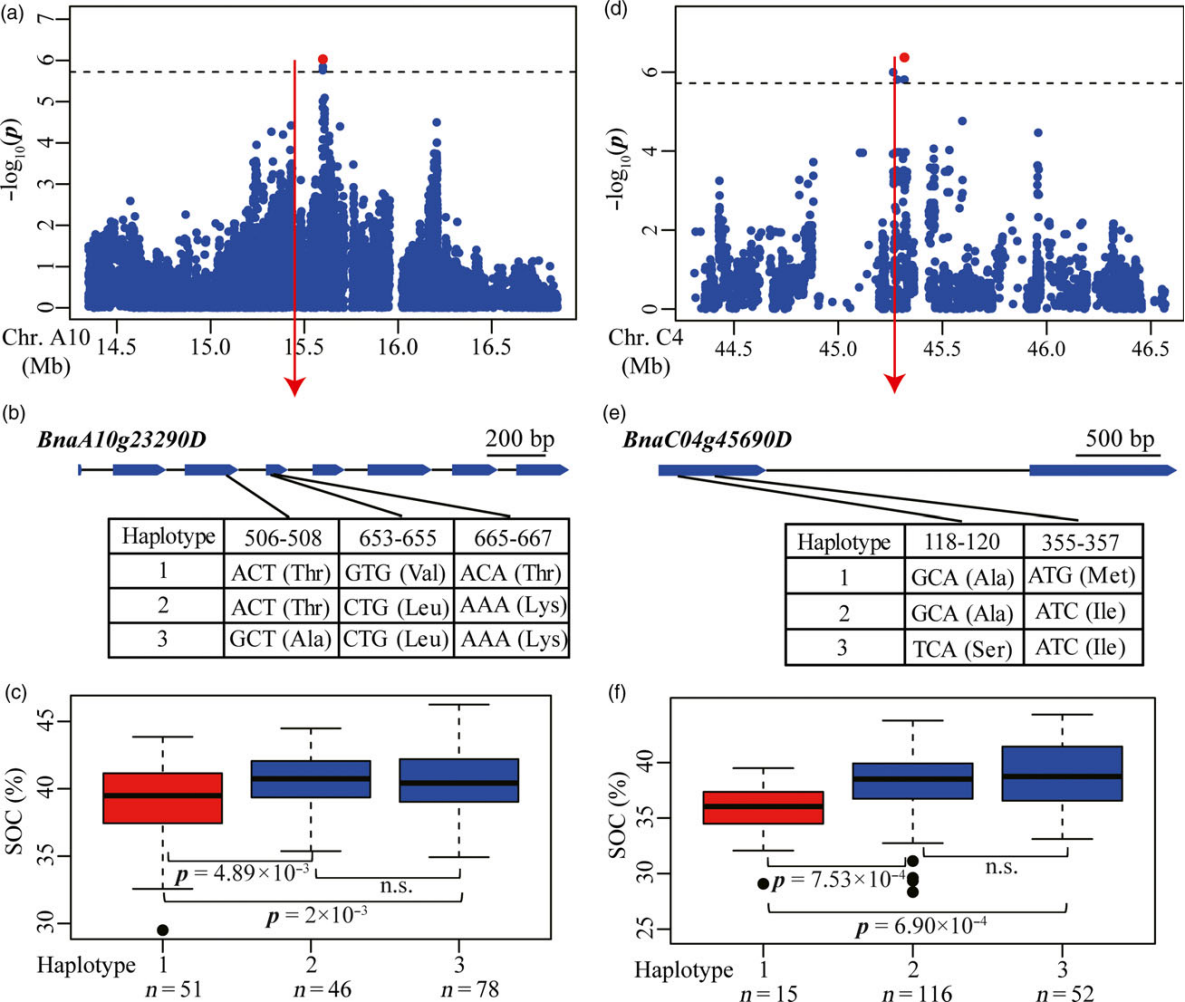
Associated loci and candidate genes for SOC on chromosome A10 and C4. (a, d) Regional Manhattan plot surrounding the peak signals on chromosome A10 in 2010 (a) and C4 in 2012 (d). Red dot indicates the peak signals snp1778098 (a) and snp2649172 (d). Dashed line represents the significance threshold. (b, e) Exon–intron structure and functional variations of *BnaA10g23290D* (b) and *BnaC04g45690D* (e). Numbers indicate the positions of open reading frame from translation start site. (c, f) Boxplots for SOC based on the haplotypes of *BnaA10g23290D* (c) and *BnaC04g45690D* (f). Differences between the genotypes or haplotypes were analysed by Wilcoxon rank‐sum test. n.s. represents not significant.

Of the seventeen associated loci, eight (47.05%) overlapped with loci identified in previous studies (Table S4), supporting the high reliability of loci detected in our study. We sought candidate genes for the associated loci and identified a total of 37 genes involved in acyl lipid metabolism underlying 16 of the associated loci (Table S5). The associations between the functional variations of these candidate genes and SOC variation were also investigated in the association panel, as described above. Peak signal snp1778098 on chromosome A10 represented an associated locus detected in 2010 (Figures 2c and 6a), which co‐localized with a locus represented by a previously identified SNP Bn‐A10‐p15924755 (Liu *et al*., 2016) (Table S4). Four candidate genes (*BnaA10g23290D*,* BnaA10g23670D*,* BnaA10g23790D* and *BnaA10g23950D*) involved in acyl lipid metabolism were found at this locus (Table S5). Several nonsynonymous SNPs were found in each of *BnaA10g23290D*,* BnaA10g23790D* and *BnaA10g23950D* except for *BnaA10g23670D*. Of these, only SNPs in *BnaA10g23290D* (*BnaA10.GDPD6*) were significantly associated with SOC variation. Three confirmed nonsynonymous SNPs located in the third and fourth exons of *BnaA10g23290D* (*BnaA10.GDPD6*) formed three haplotypes (Figures 6b and S3). Oil content of inbred lines carrying haplotype 1 (38.84 ± 3.04%) was significantly lower than lines carrying haplotype 2 and haplotype 3 (40.57 ± 2.01% and 40.63 ± 2.31%) (*P *=* *4.89 × 10^−3^ and 2.00 × 10^−3^) (Figure 6c), suggesting that the variations were significantly associated with seed oil content. The homolog of *BnaA10g23290D* in *Arabidopsis* encodes a member of the glycerophosphodiester phosphodiesterase (GDPD) family, which hydrolyses glycerophosphodiesters into sn‐glycerol‐3‐phosphate (G‐3‐P) that is a primary substrate for triacylglycerol assembly in oilseeds (Baud and Lepiniec, 2010; Cheng *et al*., 2011). These results demonstrated that *BnaA10g23290D* (*BnaA10.GDPD*) is the most likely candidate gene for the association locus represented by the peak signal snp1778098.

A further associated locus was represented by the peak signal snp2649172 on chromosome C4 (Figure 6d). This locus was detected in the 2012 environment and also co‐localizes with a previously reported associated signal Bn‐scaff_16888_1‐p1561387 (Liu *et al*., 2016) (Figure 2c; Table S4). Seven candidate genes involved in acyl lipid metabolism were found in at this locus (Table S5). Several confirmed nonsynonymous SNPs were found in each of *BnaC04g45690D* (*BnaC4.GPAT*), *BnaC04g45790D* (*BnaC4.LTP2*) and *BnaC04g45800D* (*BnaC4.LTP1*) (Figure S3). Two nonsynonymous SNPs in the first exon of *BnaC04g45690D* (*BnaC4.GPAT*) formed three haplotypes (Figure 6e). Oil content in inbred lines carrying haplotype 1 (35.57 ± 2.83%) was lower than that in lines carrying haplotype 2 and haplotype 3 (38.18 ± 2.72% and 38.93 ± 2.88%, respectively) (*P *=* *7.53 × 10^−4^ and 6.90 × 10^−4^) (Figure 6f), indicating that these variations were significantly associated with oil content. *BnaC04g45690D* (*BnaC4.GPAT*) encodes glycerol‐3‐phosphate acyltransferase involved in triacylglycerol assembly in oilseeds, which could affect seed oil content in *Arabidopsis* (Baud and Lepiniec, 2010) and has been identified as a candidate gene associated with kernel oil content in maize (Li *et al*., 2013). In addition, one nonsynonymous SNP was found in the first exon of *BnaC04g45790D* (*BnaC4.LTP2*) (Figure S6b). Inbred lines carrying GG genotype had higher oil content than lines carrying TT genotype (*P *=* *2.46 × 10^−3^) (Figure S6c). Two nonsynonymous SNPs in the first exon of the *BnaC04g45800D* (*BnaC4.LTP1*) formed two haplotypes (Figure S6d). Inbred lines carrying haplotype 1 showed lower oil content than lines carrying haplotype 2 (*P *=* *4.35 × 10^−4^) (Figure S6e). These observations suggest that variations in *BnaC04g45790D* (*BnaC4.LTP2*) and *BnaC04g45800D* (*BnaC4.LTP1*) were also significantly associated with oil content. The homologs of these two genes in *Arabidopsis* were predicted to encode lipid transfer proteins that are involved in lipid transfer between membranes (Li‐Beisson *et al*., 2010). In summary, these three genes are proposed as being responsible for the indicated peak signals and causal genes for variations of SOC in *B. napus*.

## Discussion

With the rapid development and low cost of next‐generation sequencing, GWAS based on millions of markers has become a powerful and popular approach in dissecting the genetic architecture of complex agronomic traits for crops such as rice (Huang *et al*., 2010; Yano *et al*., 2016) and maize (Li *et al*., 2013; Xiao *et al*., 2017). In our previous study, we evaluated the pattern of linkage disequilibrium in the 189 association panels using 10 343 markers generated by double‐digested restriction‐site associated DNA (ddRAD) and carried out an initial dissection of the genetic determinants of oil content as test of GWAS in rapeseed (Wu *et al*., 2016). In this study, direct resequencing of 238 rapeseed lines provided a high‐resolution genomic variation map consisting of 4 542 011 SNPs and 628 666 INDELs (Figure 1 and Table S2). This enabled us to perform a more exhaustive GWAS for three important seed‐quality traits including EAC, GSC and SOC, using 3 824 552 SNPs and INDELs in the same rapeseed association panel (Wu *et al*., 2016). The high marker density helps identifying numerous variations within genes, which facilitated identification of a more complete set of candidate genes responsible for each trait. The significant loci and candidate genes uncovered in the current study now provide us a comprehensive understanding of the genetic architecture of these three important seed‐quality traits, and this in turn provides a stronger evidence for genetic improvement of seed quality in rapeseed.

In this study, six loci were identified to be associated with EAC, 49 with GSC and 17 with SOC, distributed across the whole A and C subgenomes of *B. napus* (Figure 2; Table S4). The number of QTL detected by GWAS was significantly higher than that identified in previous single segregation and association studies (Harper *et al*., 2012; Li *et al*., 2014; Liu *et al*., 2016; Lu *et al*., 2014; Qu *et al*., 2015). Notably, the distribution patterns of EAC and GSC were similar (Figure S1), which is consistent with modern co‐selection for low levels of these two traits which define ‘canola quality’ or ‘double‐low’ rapeseed. However, it was found that the number of associated loci for GSC is much higher than that for EAC, which is consistent with a more complex genetic architecture for GSC compared with EAC (Review in introduction). This is likely to be due to the influence of multiple factors affecting total seed GSC, including the biosynthesis of aliphatic, benzenic and indolic glucosinolates, and the breakdown and transportation of glucosinolates (Borpatragohain *et al*., 2016; Nour‐Eldin *et al*., 2012; Wittstock and Burow, 2010), which was also supported by the candidate genes identified in this study (Figures 5 and S5). In contrast, erucic acid is a single compound, which is extended from a single well‐defined substrate (oleic acid) by FAE1. Moreover, QTL analysis has shown that loci for EAC and SOC on chromosome A8 and C3 overlapped in several but not all populations (Delourme *et al*., 2006; Jiang *et al*., 2014; Li *et al*., 2014; Liu *et al*., 2016; Qiu *et al*., 2006). In our association panel, the two *FAE1* genes for EAC were identified at chromosome A8 and C3 (Figure 4), whereas no loci for SOC were detected at these positions, which was also revealed in previous association studies (Li *et al*., 2014; Liu *et al*., 2016). Thus, the detection of these two loci for SOC appears to be sensitive to the genotype composition of the panels used to date, many of which may be skewed in allele composition at these secondary domestication loci. Another reasonable explanation is that two independent but linked genes responsible for EAC and SOC may be located within these two regions (Delourme *et al*., 2006; Jiang *et al*., 2014), which may also be supported by an observed weak correlation between these two traits in our panel (*r* = 0.15, *P *=* *0.05) and other population (Bhardwaj and Hamama, 2000).

In this study, we tried to identify causal sequence variants contributing to the observed phenotypic variation at loci associated with each trait. This involved first extracting genes expected to be involved in the pathway of fatty elongation, glucosinolate metabolism and acyl lipid metabolism, associated with each of EAC, GSC and SOC, respectively, based on information derived from annotation of *Arabidopsis* homologs. The most likely candidate genes were then uncovered by identifying functional polymorphisms, by testing the correlation between functional polymorphisms and phenotypic variation. The causal variations in genes *BnaA8.FAE1* and *BnaC3.FAE1* at the two major QTLs for EAC on chromosome A8 and C3 had previously been identified and validated to determine erucic acid content (Han *et al*., 2001; Wang *et al*., 2010b; Wu *et al*., 2008). By whole‐genome resequencing in our study, these were also found with direct identification of causal variations (Figure 4). Three orthologs of *MYB28* on chromosomes A9, C2 and C9 had previously been proposed as the candidate genes underlying three loci for GSC (Li *et al*., 2014). In this study, we also detected these three loci on chromosomes A9, C2 and C9 (Figures 5 and S4), and identified an INDEL in the *BnaC9.MYB28* on chromosome C9 as the most likely causal variation (Figure 5).

GWAS using whole‐genome sequencing data and functional polymorphisms in gene regions is expected to facilitate identification of genes associated with agronomic traits as well as sequence variations for genes correlated with phenotypic variation (Yano *et al*., 2016). Using this approach, we identified additional novel candidate genes associated with GSC and SOC. For GSC, the annotation of 27 putative candidate genes at fifteen associated loci implicated roles not only in the biosynthesis of aliphatic, benzenic and indolic glucosinolates, but also in the breakdown and transportation of glucosinolates (Table S6) (Borpatragohain *et al*., 2016; Nour‐Eldin *et al*., 2012; Wittstock and Burow, 2010). Association analysis between functional variations in gene regions and GSC variation revealed that seed GSC was highly correlated with four genes including *BnaA9.APK1*,* BnaA5.NSP1*,* BnaA6.MYB118* and *BnaA9.AOP3* (Figures 5 and S5). Seed oil in oilseed crops is mainly composed of triacylglycerol (TAG) fatty acids, and regulation of oil production involving many genes has been studied comprehensively in *Arabidopsis* (Baud and Lepiniec, 2010; Li‐Beisson *et al*., 2010). In this study, we identified 37 candidate genes involved in acyl lipid metabolism at sixteen associated loci for SOC in rapeseed (Table S5). Furthermore, it was confirmed that SOC variation was highly correlated with functional variations in four genes including *BnaA10.GDPD*,* BnaC4.GPAT*,* BnaC4.LTP2* and *BnaC4.LTP2* (Figures 6 and S6). These genes represent excellent a priori candidates for follow‐up analyses of causal polymorphisms and ultimately confirmed by transformation. However, for some associated loci, we failed to detect correlation between functional variations in gene regions and phenotypic variation in candidate genes. This may be due to phenotypic variation being determined by the transcriptional level of candidate genes affected by sequence polymorphisms in *cis*‐regulatory regions. For instance, a co‐expressed gene module containing 250 unigenes has been found to be highly correlated with the glucosinolate content of seeds in rapeseed (Harper *et al*., 2012). In maize, one‐third of the genes in complex coexpression networks has been found to affect oil content through transcriptional regulation (Li *et al*., 2013). Alternatively, some unknown genes or genomic components involved in *trans*‐regulatory or epistatic interactions may be responsible for the indicated associated loci.

Evidence from previous studies indicates that SOC could be increased by accumulation of favourable allele of associated loci with minor effects in crops (Li *et al*., 2013; Liu *et al*., 2016). In our study, we also observed a remarkable positive correlation between the variance of SOC and the number of favourable alleles at associated loci with minor effects (Figure 3e). In contrast, there was a remarkable negative correlation between the number of favourable allele at associated loci and the variances of EAC and GSC (Figure 3c,d), suggesting that EAC and GSC could be further reduced by accumulating favourable alleles, although they are both controlled by major QTLs coupled with several minor QTLs. This is consistent with the secondary domestication (Wang *et al*., 2017), whereby ‘double‐low’ canola *B. napus* was selected following introgression of alleles from two well‐defined cultivar sources Liho (low EAC) and Bronowski (low GSC) (Fu and Gugel, 2010; Klassen *et al*., 1987). Our results also suggest that selecting the favourable allele for each of the significant loci detected by GWAS may provide a straightforward means for breeding elite cultivars with robust lower EAC and GSC and higher SOC. Notably, there is evidence that loci associated with EAC and SOC have already undergone selection in rapeseed breeding programmes with favourable allele frequencies of all associated loci being much higher (Figure 3a). In contrast, the favourable allele frequency of many loci associated with GSC, some of which had a greater effect on GSC, was relatively low compared with EAC and SOC (Figure 3a). These loci may not yet have been utilized and could be very useful for further reducing GSC in rapeseed breeding. But, any process of genomic selection needs to take into account issues of linkage drag, where deleterious alleles of genes that affect independent traits may be in coupling with beneficial alleles. However, it should be possible to pyramid favourable alleles of these loci in single target lines through marker‐assisted selection (MAS). Our association panel showed that to date only a few inbred lines contained all favourable alleles (Figure 3c–e) and so there is potential to further improve these traits by pyramiding all favourable alleles in single line.

## Experimental procedures

### Plant materials and phenotyping

A total of 238 rapeseed cultivars or inbred lines were collected from the major production areas of the world to represent the genetic diversity of *B. napus* cultivars (Table S1); 189 of the 238 cultivars or inbred lines prefixed with letter ‘g’ were employed for phenotyping and genome‐wide association mapping in this study (Table S1). The 189 cultivars or inbred lines had been well described in previous study and were classified into two main groups based on both population structure and principal component analysis: P1 group with 136 lines mainly originated from China and P2 group with 53 lines mainly originated from Europe, Canada and Australia (Wu *et al*., 2016). Field trials were performed using a random block design with three replications in each environment from 2009 to 2012 at Huazhong Agricultural University, Wuhan, China, in the winter–spring growing seasons. Each line was grown in a plot of two rows with ten plants in each row, with 20 cm apart within row and 30 cm between rows. Five plants in the middle of each plot were harvested for trait measurements. EAC was measured by gas chromatography (Agilent 7890) and expressed as a percentage of the total fatty acid in seeds as described previously (Burns *et al*., 2003). Total GSC of seeds was estimated by a Foss NIRSystems 5000 near infrared reflectance spectroscope (Harper *et al*., 2012). SOC was estimated by nuclear magnetic resonance using ~3‐g dry seeds (mq20, Bruker) (Borisjuk *et al*., 2011). The calibration was built using six inbred lines with SOC ranging from 28% to 50%. The correlation of calibration is 0.993, with slope of 0.430 and intercept of 1.572.

### Genome sequencing, variant identification and annotation

Total genomic DNA was isolated from fresh young leaf tissues using the CTAB method; 1 μg of genomic DNA was sheared using an ultrasonic Biorupture (Diagenode; Liege, Belgium). Short‐insert libraries were constructed according to the manufacturers' instructions (Illumina) and then sequenced using Illumina Hiseq2000 platform to produce 100‐bp paired‐end (PE100) reads. The sequence data for cultivar ‘Zhongshuang 11’ (ZS11) were *in silico* generated from the assembled scaffold sequences (Sun *et al*., 2017).

The genome assembly of *B. napus* cultivar Darmor‐'*bzh*’ v4.1 (Chalhoub *et al*., 2014) was used as a reference for mapping of all short reads from the diversity analysis. The PE100 reads from individuals were aligned to the reference genome using the Burrows‐Wheeler Alignment tool (BWA, version 0.7.0), with a maximum of three mismatches and one gap of 1 to 10 bp at each end. Only the PE100 reads with both ends located within a genomic region <1000 bp were kept for subsequent analysis. The alignment results in SAM format were converted to the BAM format using SAMtools (version 0.1.18). Duplicated reads caused by PCR in the process of library construction were identified and removed using Picard package (version 1.91). The reads with gaps were realigned using the local realignment tool in Genome Analysis Toolkit (GATK, version 2.4). SNP and INDEL (1–10 bp) calling for all 238 accessions was performed using the GATK. The nonsingleton variants, which were present in two or more lines, were retained as ‘raw variants’ for further analysis. To obtain high‐quality variants, raw variants were further filtered using the following criteria: (i) relative heterozygosity (*H*_*R*_) less than 0.2 (Wu *et al*., 2016), which was used to discriminate simple SNPs/INDELs (only single allele is present in most of accessions) from hemi‐SNPs/INDELs and interhomoeologue polymorphisms (IHPs) (Trick *et al*., 2009); (ii) the percentage of missing genotype of one variant is less than 60%. Finally, missing genotypes were imputed using Beagle software (version 3.3.2). To evaluate the imputation accuracy, 10 genomic regions each containing 10 000 consecutive variants were selected and 1% of the known genotypes were masked randomly. After five repeats of mask and imputation, the imputation accuracy was estimated to 98.89%, which was similar to that obtained previously (Huang *et al*., 2010).

The identified SNPs and INDELs were annotated using custom Perl scripts and the ANNOVAR program (Wang *et al*., 2010a), using the ‘Darmor‐*bzh*’ gene model v5.0. The output of ANNOVAR was assigned to three categories: (i) genic region: ‘exonic’, ‘intronic’, ‘splicing’, ‘UTR5’ and ‘UTR3’; (ii) upstream/downstream region: ‘upstream’, ‘downstream’ and ‘upstream&downstream’; (iii) intergenic region.

### Statistical analysis

Broad‐sense heritability (*H* ^2^) for each trait in single and across environment was calculated as $H^{2}=\delta_{G}^{2}/\left(\delta_{G}^{2}+\delta_{e}^{2}/r\right)$, and $H^{2}=\delta_{G}^{2}/\left(\delta_{G}^{2}+\delta_{\mathrm{GE}}^{2}/n+\delta_{e}^{2}/nr\right)$, respectively, where $\delta_{G}^{2}$ is the genetic variance, $\delta_{\mathrm{GE}}^{2}$ is the variance due to *G* × *E* interaction, $\delta_{e}^{2}$ is the residual error, *n* is the number of environments, and *r* is the number of replicates within environment. The estimates of $\delta_{G}^{2}$, $\delta_{\mathrm{GE}}^{2}$ and $\delta_{e}^{2}$ were obtained from analysis of variance (ANOVA) using the lmer function in lme4 package in R environment in R program (Team, 2012). The average trait values of inbred lines from replicates in each year were used for phenotypic analysis and association mapping in single environment. Best linear unbiased predictor (BLUP) of line effect was estimated using R package ‘lme4’ (Bates *et al*., 2014). The BLUP values of each trait were used for phenotypic analysis across all environments. The normality test was performed (*P* < 0.05) using the R function ‘shapiro.test’ (Team, 2012).

### Genome‐wide association mapping

High‐quality SNPs and INDELs with minor allele frequency (MAF) >0.05 in the association panel were used for GWAS. GWAS was performed for each trait using a mixed linear model (MLM) in genome‐wide efficient mixed model association (GEMMA) software (Zhou and Stephens, 2012). MLM was coupled with estimated relatedness matrix as a random effect, which was estimated by GEMMA. The relative kinship in the association panel had been well described previously (Xiao *et al*., 2012). The effective number of independent markers (N) was calculated using the GEC tool (Li *et al*., 2012), and the suggestive *P* value (1/N) was set as the threshold as described previously (Yang *et al*., 2014). The Manhattan and quantile–quantile (Q–Q) plots of GWAS results were generated in R program. To display the unique associated loci for each trait, all consecutive significant markers with pairwise linkage disequilibrium (LD) cut‐off value of *r* ^2^ > 0.5 were integrated into a single locus, using marker in peak signal (lowest *P*‐value) as the proxy. The phenotypic variation explained (PVE) of single significant locus was estimated by ANOVA as described previously (Li *et al*., 2013). Total variance explained (*R* ^2^) was estimated using all proxy marker of each trait in R package *leaps*. The adjusted *R* ^2^ was regarded as the PVE of all significant loci.

### Identification of candidate genes

The gene models of *B. napus* annotation v5 (Chalhoub *et al*., 2014) and *Arabidopsis* annotation TAIR10 (http://www.arabidopsis.org/) were used to uncover the candidate genes underlying identified loci. The gene models of the *B. napus* genome were aligned to the protein database of *Arabidopsis* using Basic Local Alignment Search (BLAST) with *E*‐value <10^−30^. The best hit genes in *Arabidopsis* were regarded as the corresponding homologous genes of queries in *B. napus*. Meanwhile, the identified genes involved in the pathways of acyl lipid and glucosinolate metabolism in *B. napus* genome were obtained from a previous study (Chalhoub *et al*., 2014). To identify the putative candidates associated with each trait, the regions of 300 kb up/downstream of the peak signals were selected for further analysis, as described previously (Xu *et al*., 2016).

### Validation of variations in the candidate genes

Sequences of candidate genes were obtained from the *B. napus* cultivar Darmor‐’*bzh*’ v4.1 reference sequence (Chalhoub *et al*., 2014). Primers were designed using Primer3 software (http://primer3.sourceforge.net/) based on the flanking sequences of each variant and the specificity of the primers were checked using e‐PCR software. PCRs were performed according to instructions using KOD‐plus‐Neo (Catalog KOD‐401; TOYOBO), with predenaturation at 94 °C for 3 min followed by 35 cycles at 98 °C for 10 s, 60 °C for 30 s, 68 °C for 35 s and a final extension at 68 °C for 5 min. PCR products were directly sequenced using 3730 sequencers (ABI). All primers were listed in Table S7.

## Availability of data

The Illumina sequence data supporting the conclusions of this study are available in the National Center for Biotechnology Information (NCBI) Sequence Read Archive (SRA) repository with the accession number of SRP067370 and SRP125656.

## Author contributions

K.L. designed and supervised the study. B.W., Z.W., Z.L. and H.L. analysed the data. Z.W., J.H., Y.X., D.C and J.W. performed the field trials. Q.Z. and H.L. performed the genome sequencing. B.W., Z.W., Y.X., G.J.K., H.L. and K.L. wrote the manuscript.

## Supporting information


**Figure S1** Phenotypic distributions of the three seed‐quality traits across each year.
**Figure S2** Quantile‐quantile plots for the three seed‐quality traits.
**Figure S3** Validation of functional variants in the candidate genes.
**Figure S4** Associated loci for glucosinolate content (GSC) on chromosome A9 and C2.
**Figure S5** Associated loci and candidate genes for glucosinolate content (GSC) on chromosome A5, A6 and A9.
**Figure S6** Associated loci and candidate genes for seed oil content (SOC) on chromosome C4.

**Table S1** Summary of 238 varieties and resequencing data.
**Table S2** Distribution of SNPs and INDELs on 19 chromosomes of *B. napus*.
**Table S3** The annotation of SNPs and InDels in *B. napus*.
**Table S4** Summary of genome‐wide significant association signals.
**Table S5** The candidate genes for the associated loci detected by GWAS.
**Table S6** Summary of pathways of the candidate genes for GSC.
**Table S7** List of the primers used in this study.
