# Supplementary material for: Dissection of the genetic architecture of three seed‐quality traits and consequences for breeding in Brassica napus
Source: Plant Biotechnol J. 2018 Jan 25;16(7):1336–48. doi: 10.1111/pbi.12873 (PMC5999192; doi:10.1111/pbi.12873)
Supplement: Supplementary file 1 — Figure S1 Phenotypic distributions of the three seed‐quality traits across each year. Figure S2 Quantile‐quantile plots for the three seed‐quality traits. Figure S3 Validation of functional variants in the candidate genes. Figure S4 Associated loci for glucosinolate content (GSC) on chromosome A9 and C2. Figure S5 Associated loci and candidate genes for glucosinolate content (GSC) on chromosome A5, A6 and A9. Figure S6 Associated loci and candidate genes for seed oil content (SOC) on chromosome C4. [file PBI-16-1336-s001.docx]

**Fig. S1** Phenotypic distributions of the three seed-quality traits across each year.

**
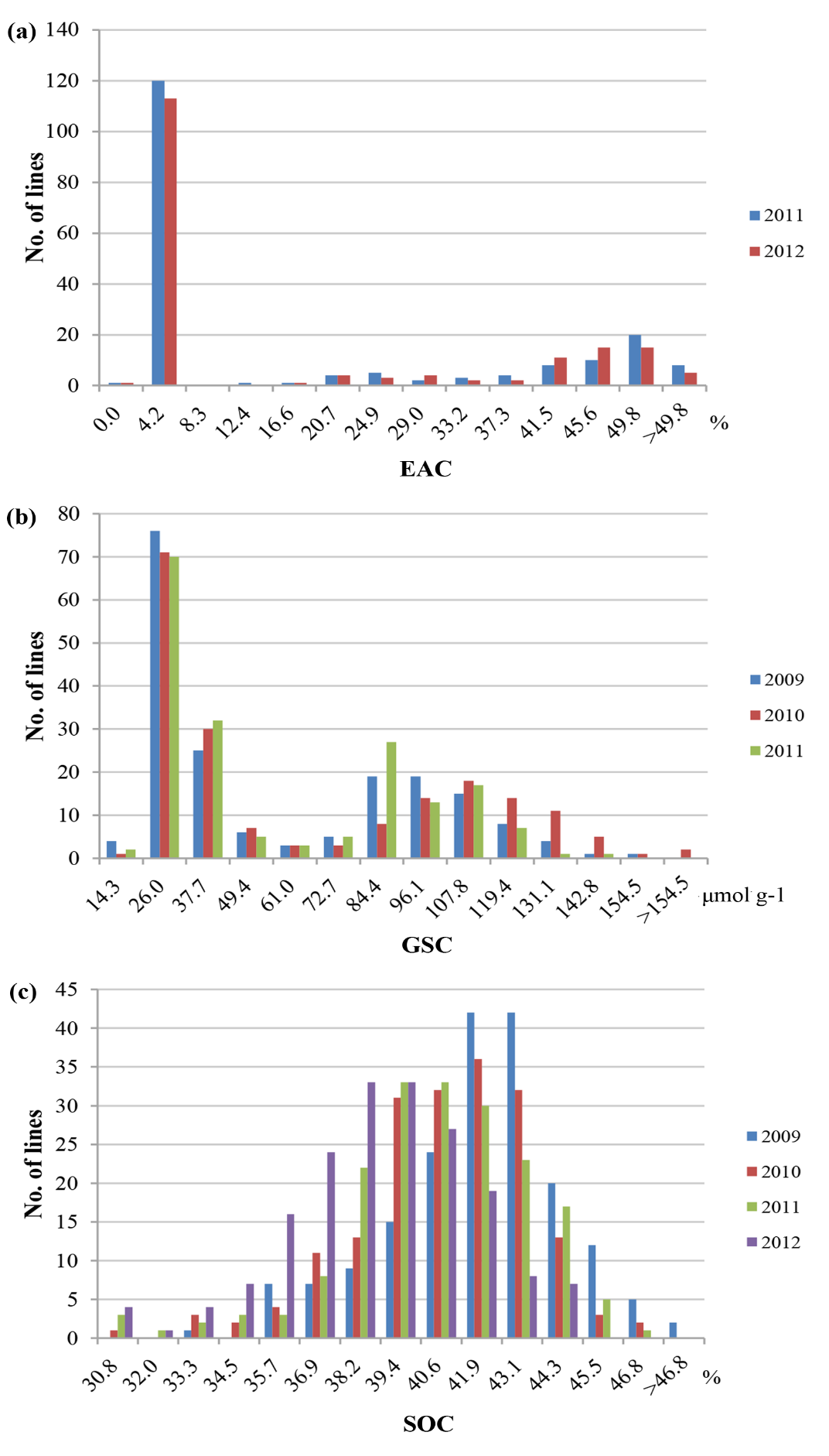
**

**Fig. S2** Quantile-quantile plots for the three seed-quality traits. (a) EAC in 2011 and 2012. (b) GSC in 2009, 2010 and 2011. (c) SOC in 2009, 2010, 2011 and 2012. ­

**
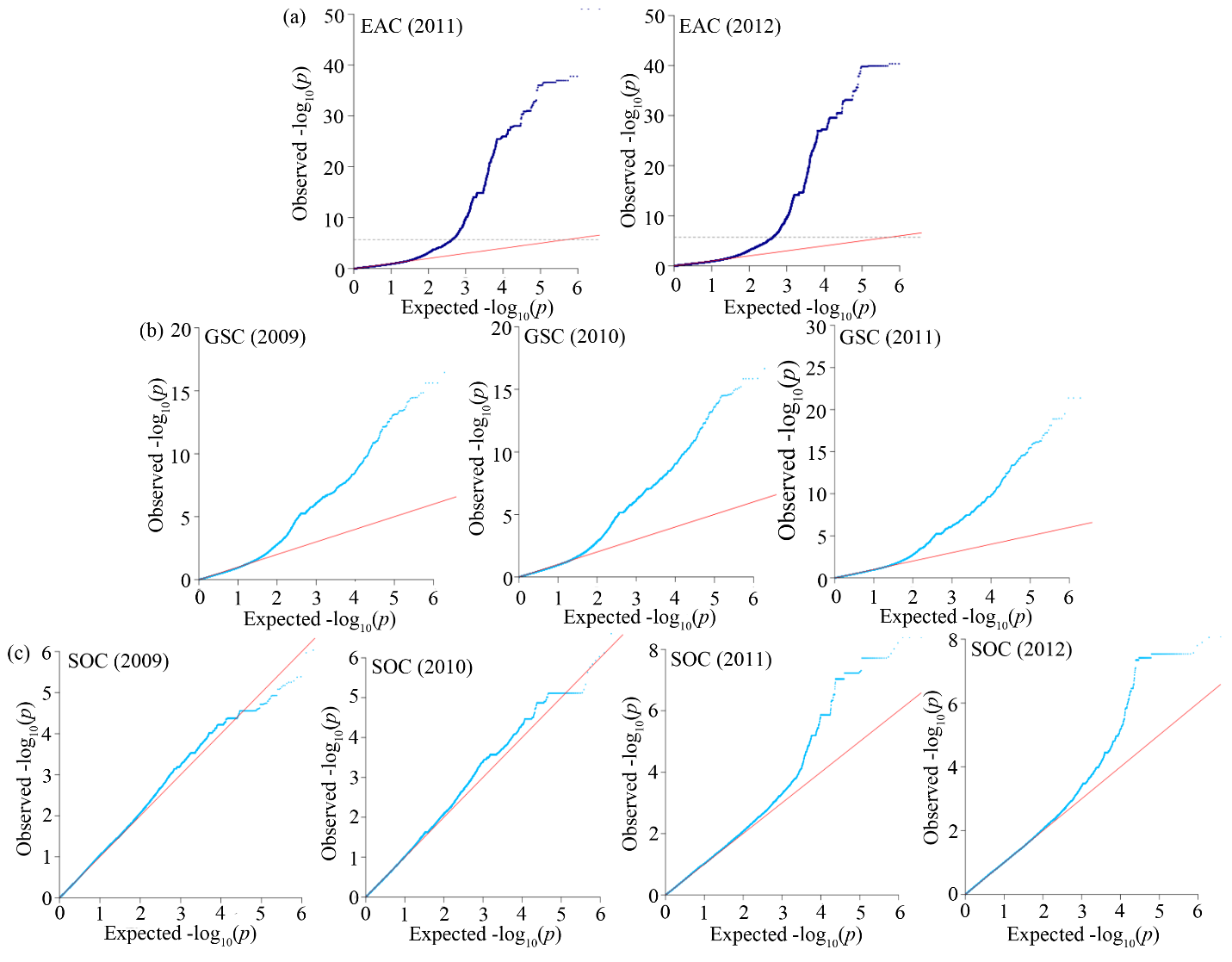
**

**Fig. S3 Validation of functional variants in the candidate genes.** The variations were highlighted in black.

**
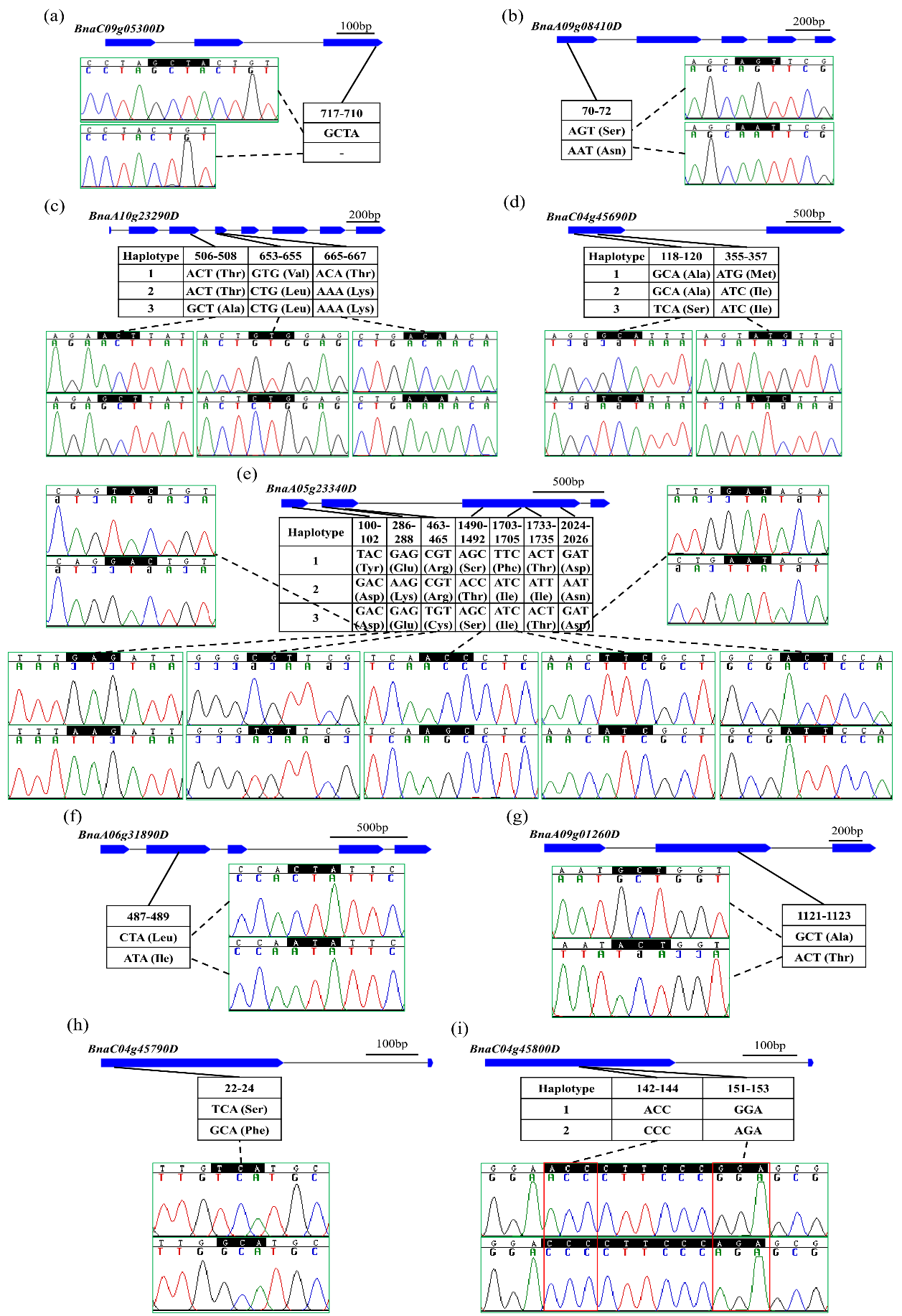
**

**Fig. S4 Associated loci for glucosinolate content (GSC) on chromosome A9 and C2**. (a, c) Regional Manhattan plot surrounding the peak signals on chromosome A9 (a) and C2 (c) in 2012 (d). Red dot indicates the peak signals snp1438406 (a) and indel338983 (c). Horizontal dashed line represents the significance threshold. (b, d) Regional alignment of genes surrounding *Bra035929* between *B. rapa* and *B. napus* (b), and *Bo2g161590* between *B. oleracea* and *B. napus* (d). The information of homologs was obtained from previous study (Chalhoub et al., 2014).

**
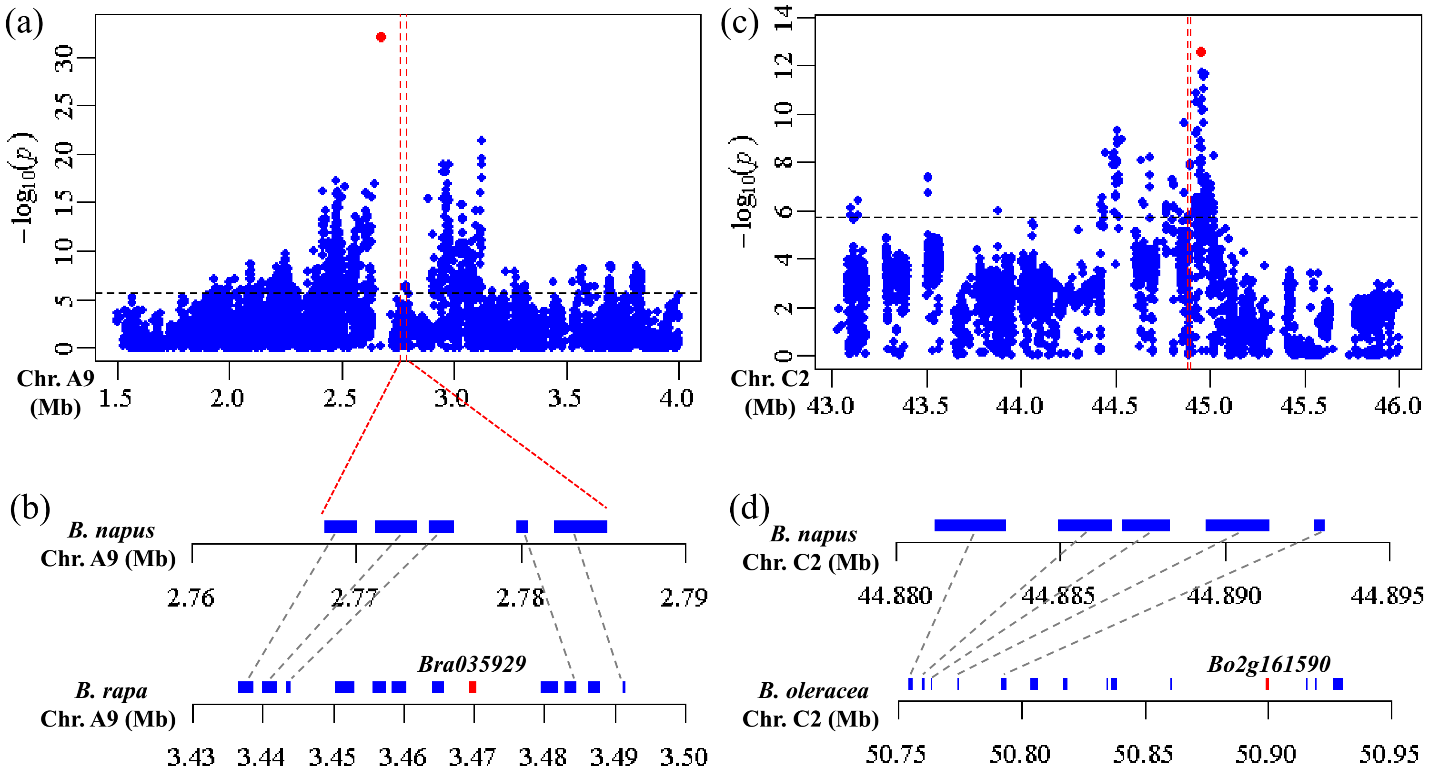
**

**Fig. S5 Associated loci and candidate genes for glucosinolate content (GSC) on chromosome A5, A6 and A9**. (a, d and g) Regional Manhattan plot surrounding the peak signals on chromosome A5 (a), A6 (d) and A9 (g) in 2011. Red dot indicates the peak signal snp846562 (a), snp1064558 (d) and snp1431371 (g). Horizontal dashed line represents the significance threshold. (b, e and h) Exon-intron structure and functional variation of *BnaA05g23340D* (b), *BnaA06g31890D* (e) and *BnaA09g01260D* (h). Numbers indicate the positions of open reading frame from the translation start site. (c, f and i) Boxplots for GSC based on the haplotypes of *BnaA05g23340D* (c) and genotypes of *BnaA06g31890D* (f) and *BnaA09g01260D* (i). Differences between the genotypes were analyzed by Wilcoxon Rank-Sum test. n.s. represents not significant.

**
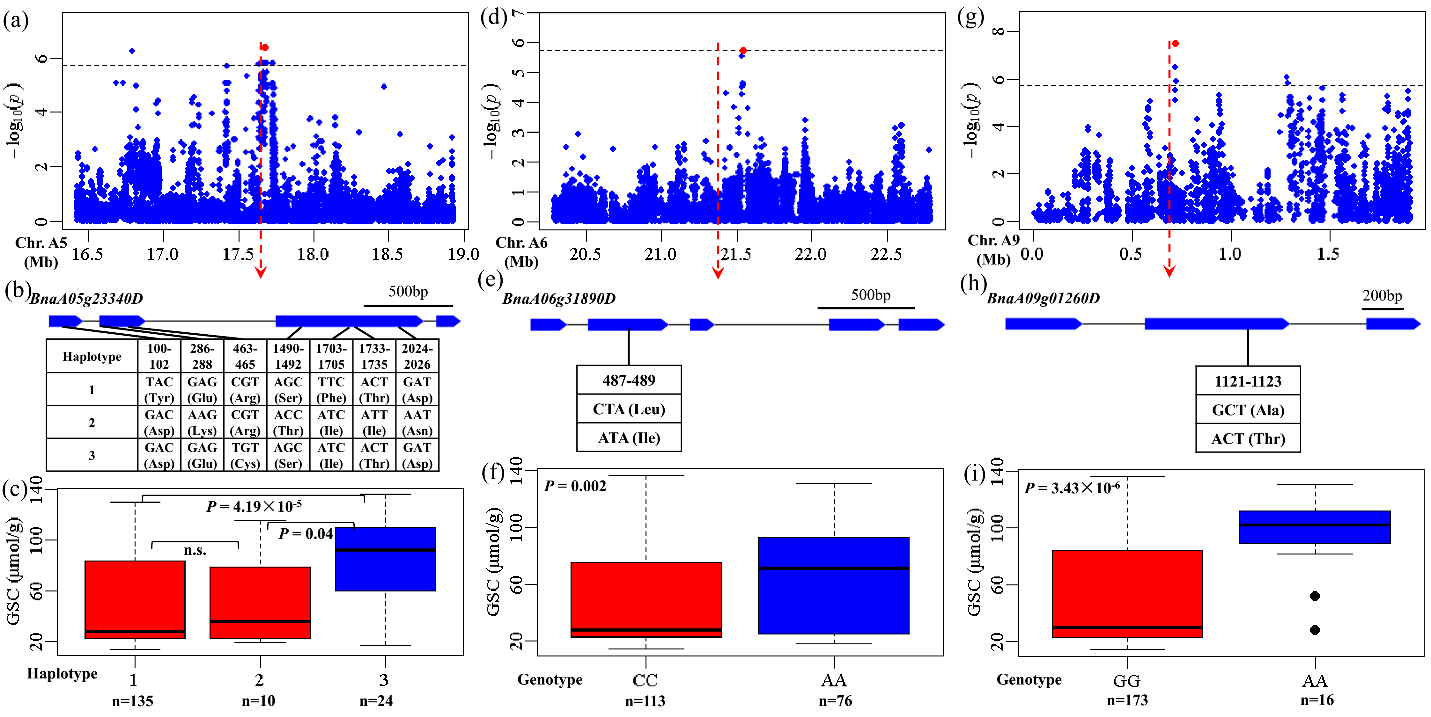
**

**Fig. S6 Associated loci and candidate genes for seed oil content (SOC) on chromosome C4**. (a) Regional Manhattan plot surrounding the peak signals on chromosome C4 in 2012 (d). Red dot indicates the peak signal snp2649172 (d). Horizontal dashed line represents the significance threshold. (b, d) Exon-intron structure and functional variations of *BnaC04g45790D* (b) and *BnaC04g45800D* (d). Numbers indicate the positions of open reading frame from the translation start site. (c, e) Boxplots for SOC based on the genotypes of *BnaC04g45790D* (c) and haplotypes of *BnaC04g45800D* (e). Differences between the genotypes or haplotypes were analyzed by Wilcoxon Rank-Sum test.

**
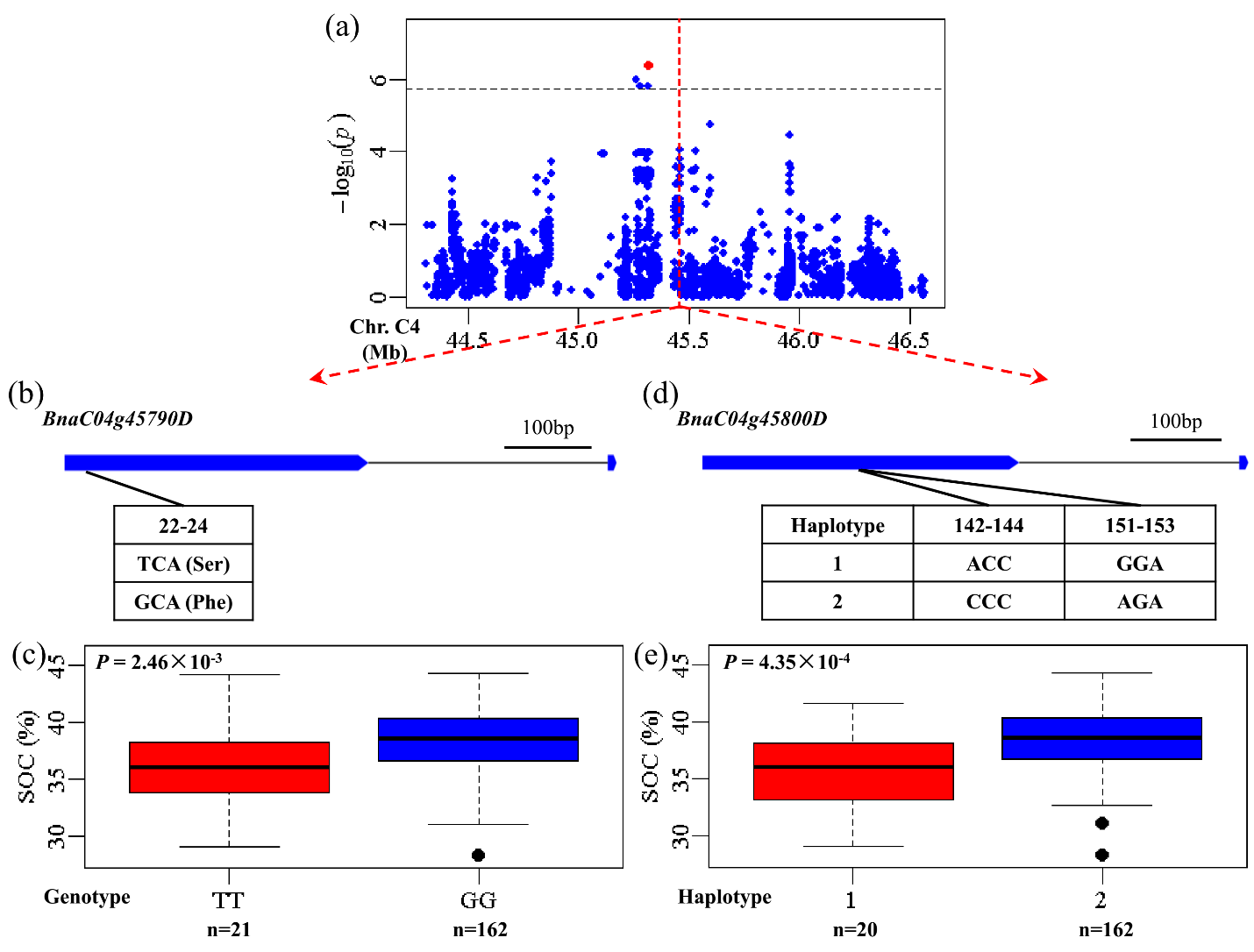
**
